# A subcomplex of human mitochondrial RNase P is a bifunctional methyltransferase—extensive moonlighting in mitochondrial tRNA biogenesis

**DOI:** 10.1093/nar/gks910

**Published:** 2012-10-05

**Authors:** Elisa Vilardo, Christa Nachbagauer, Aurélie Buzet, Andreas Taschner, Johann Holzmann, Walter Rossmanith

**Affiliations:** Center for Anatomy and Cell Biology, Medical University of Vienna, 1090 Vienna, Austria

## Abstract

Transfer RNAs (tRNAs) reach their mature functional form through several steps of processing and modification. Some nucleotide modifications affect the proper folding of tRNAs, and they are crucial in case of the non-canonically structured animal mitochondrial tRNAs, as exemplified by the apparently ubiquitous methylation of purines at position 9. Here, we show that a subcomplex of human mitochondrial RNase P, the endonuclease removing tRNA 5′ extensions, is the methyltransferase responsible for m^1^G9 and m^1^A9 formation. The ability of the mitochondrial tRNA:m^1^R9 methyltransferase to modify both purines is uncommon among nucleic acid modification enzymes. In contrast to all the related methyltransferases, the human mitochondrial enzyme, moreover, requires a short-chain dehydrogenase as a partner protein. Human mitochondrial RNase P, thus, constitutes a multifunctional complex, whose subunits moonlight in cascade: a fatty and amino acid degradation enzyme in tRNA methylation and the methyltransferase, in turn, in tRNA 5′ end processing.

## INTRODUCTION

Transfer RNAs (tRNAs) are the essential adaptors in the decoding of messenger RNAs (mRNAs) by the ribosome; thus, their faithful biogenesis is crucial for cell survival. tRNAs are transcribed as precursors and undergo several steps of nucleolytic processing, nucleotide addition, editing and nucleotide modification (1). tRNAs are the most extensively modified type of cellular RNA, and to date, ∼100 different modifications have been identified (2,3). The extent of resources invested by cells in maintaining and regulating all the pathways involved in tRNA modification highlights their importance (1). Modifications in the anticodon region expand the decoding capacity and improve translational fidelity and are often essential. In contrast, the effects of modifications in the tRNA core are less obvious and still poorly understood. Most core-modifications are pseudouridinylations and different kinds of methylations at several nucleotide positions, and they are thought to stabilize the 3D structure of tRNAs by favouring or impeding specific base interactions (4,5). The lack of a single of these modifications often has unremarkable effects, but the removal of more than one can structurally and metabolically destabilize tRNAs, and thereby preclude cell survival (6).

The mitochondria of most Eukarya have a dedicated set of tRNAs encoded in the organellar genome. Mitochondrial tRNAs [(mt)tRNAs] of animals are peculiar in sequence and structure, and they are often referred to as ‘bizarre’, to underline their deviation from the structural consensus conserved in other tRNAs (7,8). The best studied mammalian (mt)tRNAs tend to have a lower structural stability resulting from a bias in nucleotide composition, non-canonical secondary structures and a lack of conserved tertiary interactions (8–12). Modifications of these (mt)tRNAs, although less in number, seem to be more important and, in some cases, crucial to achieve correct folding, and the impairment of even single modifications was reported to cause mitochondrial disease (4,5,12).

**Figure 1.**
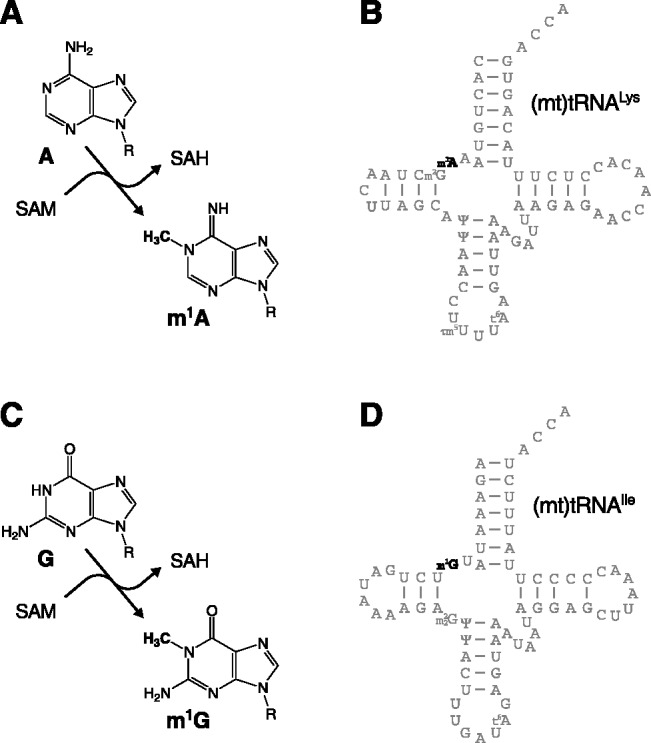
Methylation of adenosine and guanosine at position 9 of human (mt)tRNAs. (**A**) Enzymatic methylation of the *N*^1^ of adenosine using SAM as the methyl group donor and release of *S*-adenosyl homocysteine (SAH). (**B**) Cloverleaf structure and modified nucleotides of human (mt)tRNA^Lys^. (**C**) Enzymatic methylation of the *N*^1^ of guanosine. (**D**) Cloverleaf structure and modified nucleotides of human (mt)tRNA^Ile^.

A modification with particularly strong effect on tRNA folding is the *N*^1^-methylation of adenosine in position 9 (m^1^A9) of human (mt)tRNA^Lys^ (Figure 1A and B). Unmodified tRNA^Lys^ folds into a non-functional extended stem-loop; methylation of A9 impedes its base pairing with U64, and thereby shifts the equilibrium to the cloverleaf structure (13,14). Position 9 is occupied by A or G in 19 of the 22 human (mt)tRNAs and, considering the modification pattern of all currently analysed animal (mt)tRNAs, it is probably modified to m^1^A or m^1^G in all 19 (15). m^1^A9 is otherwise only found in some archaeal tRNAs, and m^1^G9 is restricted to a subset of the archaeal and eukaryal cytosolic tRNAs harbouring G9 (15,16). The ubiquitous occurrence of the two modifications in animal mitochondria suggests a more general, critical importance for the structural integrity of (mt)tRNAs. The enzyme(s) responsible for the methylation of A9 and G9 in (mt)tRNAs, however, has/have not been identified to date.

We recently identified a presumptive methyltransferase as a subunit of human mitochondrial RNase P [mtRNase P (17)]. This is the endonuclease responsible for the formation of the 5′ ends of (mt)tRNAs and the concomitant release of 5′ flanking sequences from the polycistronic mitochondrial primary transcripts. Human mtRNase P comprises three proteins, one of which has the structural features of a metallonuclease (17,18). The other two proteins do not have any obvious relationship to nucleases, and their role for mtRNase P-function is unclear. However, one of them, originally termed mitochondrial RNase P protein 1 (MRPP1), now tRNA methyltransferase 10 C (TRMT10C) according to the recent official nomenclature (see Supplementary Table S1 for nomenclature and synonyms of the proteins/genes studied in this article), is homologous to yeast Trm10p, a tRNA:m^1^G9 methyltransferase (19). Vertebrate genomes encode two further homologues of *TRM10*, but those apparently have no mitochondrial targeting sequence. None of the three human Trm10p homologues has yet been shown to have methyltransferase activity. Still, TRMT10C is a candidate for being responsible for at least m^1^G9 in human (mt)tRNAs, and possibly for m^1^A9 too. The physical association of TRMT10C with mtRNase P, moreover, suggests that methylation and cleavage are possibly also functionally connected. With this work, we, thus, sought to not only identify and characterize a modification enzyme crucial for (mt)tRNA function but also to investigate whether mtRNase P is in fact a multifunctional complex, gathering different enzymatic activities involved in tRNA maturation at different levels, and to understand the relationship among the different subunits.

## MATERIALS AND METHODS

### Expression and purification of recombinant proteins

The plasmids for the expression of N-terminally His-tagged TRMT10C and short-chain dehydrogenase/reductase 5C1 (SDR5C1) were described previously (17). TRMT10C starting with amino acid 40 and extending at its C-terminus into a tandem myc-6×His-tag was cloned into the *Nde*I/*Bam*HI sites of pET-21b(+) (Novagen). TRMT10C starting with amino acid 40 (including the stop codon) was cloned the *Nde*I/*Xho*I sites of pET-21b(+); the recombinant protein corresponds to the native mitochondrial protein. Proteinaceous RNase P (PRORP) starting with amino acid 46 was cloned into the *Nco*I/*Xho*I sites of pET-28b(+) (Novagen); the recombinant protein contains a C-terminal 6×His-tag. The plasmid for the expression of yeast Trm10p was kindly provided by Jane Jackman (19). The complete coding sequences (including the stop codon) of TRMT10A and TRMT10B were cloned into the *Nde*I/*Xho*I sites of pET-28b(+); recombinant proteins contain an N-terminal 6×His-tag and a thrombin cleavage site.

Proteins were expressed in *Escherichia coli* BL21(DE3) or Rosetta2(DE3) (native TRMT10C) as previously described (17). Bacteria were broken by sonication, and His-tagged proteins were purified on HisTrap HP columns (GE Healthcare) using an ÄKTA purifier chromatography system (GE Healthcare); buffer A [150 mM NaCl, 50 mM Tris–Cl pH 7.4, 10% glycerol, 1 mM dithiothreitol (DTT)]. Proteins were lysed, loaded and washed with 25 mM imidazole in buffer A (50 mM in case of SDR5C1 and PRORP), then with 150 mM imidazole, then with 1 M NaCl in buffer A, again with buffer A and finally, eluted with 500 mM imidazole. In case of PRORP, all buffers were based on HEPES–Na instead of Tris–Cl. Eluted PRORP was diluted 1:3 with buffer B (50 mM HEPES–Na pH 7.4, 10% glycerol, 1 mM DTT), loaded on a HiTrap SP XL column (GE Healthcare), washed with 150 mM NaCl in buffer B and eluted with 1 M NaCl.

Purified recombinant SDR5C1 was mixed with a crude bacterial lysate of native TRMT10C (50 mM imidazole in buffer A), and the TRMT10C–SDR5C1 complex was purified on a HisTrap HP column. The ratio of SDR5C1 to TRMT10C-lysate was determined empirically to yield a TRMT10C–SDR5C1 complex saturated with TRMT10C. The column was washed with 50 mM imidazole and then with 125 mM imidazole, and TRMT10C–SDR5C1 was eluted with 500 mM imidazole.

Mutations were introduced into the TRMT10C (untagged) and SDR5C1 expression plasmids by site-directed mutagenesis, using the QuickChange protocol (Stratagene). Mutant proteins were expressed, and TRMT10C–SDR5C1 complexes were purified in parallel with the wild-type using His-affinity-coated magnetic beads (Dynal) and the aforementioned lysis, washing and elution buffers. The ratio of the two proteins within the complex was identical for mutant and wild-type preparations.

The purity of all recombinant proteins and the ratio of TRMT10C to SDR5C1 in the complex were assessed by sodium dodecyl sulphate-polyacrylamide gel electrophoresis (SDS–PAGE) and Coomassie brilliant blue staining (Supplementary Figure S1). Purified proteins were snap frozen in liquid nitrogen and stored in aliquots at −80°C. Protein concentrations were calculated by the absorbance at 280 nm and the molar extinction coefficient and molecular weight; alternatively, the protein concentration was estimated by SDS–PAGE and Coomassie brilliant blue staining relative to bovine serum albumin standards.

### Preparation of mitochondrial extracts

HeLa cell mitochondrial extracts were prepared as previously described (17).

### RNA interference

Transfection of HeLa cells with small interfering RNAs (siRNAs) targeting TRMT10C, SDR5C1, or non-targeting control siRNAs, and RNA analysis by quantitative real-time reversed transcriptase-polymerase chain reaction (RT-PCR) were carried out as previously described (17).

### Preparation of tRNA substrates

(Mt)tRNA^Ile^ was transcribed from a PCR product of the tRNA linked at its 5′ end to a hammerhead ribozyme and a T7 promoter, and terminating at the 3′ end with the CCA sequence. All position 9-labelled tRNA substrates were prepared by ‘splint-guided’ two-way ligation of a synthetic RNA corresponding to the first eight nucleotides of the respective tRNA (prepared by solid-phase synthesis) to a 5′ end-labelled *in vitro* transcript of tRNA position 9–76 (20). The transcripts were prepared from PCR products comprising a T7 promoter, some polylinker plasmid sequence and a hammerhead ribozyme fused to the tRNA sequence from position 9 to the terminal CCA sequence. All these hammerhead-tRNA cassettes were assembled by overlap extension PCR, cloned in pGEM-1 (Promega) and, subsequently, PCR-amplified with a T7 promoter primer and a reverse primer terminating with the 3′-terminal CCA (21). To generate position 9-labelled pre-tRNA substrates for RNase P, a synthetic RNA corresponding to the first eight nucleotides of the respective tRNA plus 15 nucleotides of the natural 5′-flanking sequence was ligated to the incomplete 5′ end-labelled tRNA transcript. The I9 containing (cyt)tRNA^Arg-I9^ was prepared by ‘splint-guided’ three-way ligation (20) of the first eight nucleotides of (cyt)tRNA^Arg^ to nucleotides 9–17 (5′ end-labelled) and 18–76 (all three prepared by solid-phase synthesis). The precursor of (mt)tRNA^Ile^ was described previously (17). The (mt)tRNA^His^ precursor contains nucleotides 12113–12223 of the human mitochondrial genome cloned into the *Eco*RI/*Xba*I sites of pBS+ (Stratagene), and results in a tRNA transcript flanked by a 61-nucleotide leader and a 19-nucleotide trailer.

*In vitro* transcription, 5′ end labelling with [γ-^32^P]adenosine triphosphate and gel purification were carried out as previously described (22,23). tRNA fragments were ligated with the help of DNA ‘splints’ (complementary to the first 20 nucleotides or to all of the tRNA for three-way ligation) and T4 DNA ligase (20) and were purified by denaturing PAGE (22,23).

### Methyltransferase assay

Methylation and processing reactions were carried out in 50 mM Tris–Cl pH 8, 20 mM NaCl, 4.5 mM MgCl_2_, 1 mM DTT, 20 µg/ml bovine serum albumin, 20 units/ml RiboLock RNase inhibitor (Fermentas). Substrates at 3–10 nM were methylated at 30°C with 25 µM *S*-adenosyl methionine (SAM) and recombinant proteins at 40–200 nM (except otherwise indicated). Samples were withdrawn at defined intervals or at known linear-range time-points, stopped by addition of guanidinium thiocyanate and were ethanol-precipitated. Samples were dissolved in H_2_O, NH_4_–acetate pH 5.3 and nuclease P_1_ added to 60 mM and 0.1 U/µl, respectively, and the RNA was hydrolysed to completion for 1 h at 37°C. Samples were spotted on cellulose coated thin-layer chromatography (TLC) plastic sheets (Polygram CEL 300; Macherey-Nagel) and developed in solvent B, or first in solvent A and then solvent B for 2D TLC (24). Dried TLC plates were subjected to storage phosphor autoradiography. Additional samples were routinely taken from methylation reactions at final time-points, stopped by mixing with one volume of formamide gel-loading buffer (22), and RNA integrity was verified by denaturing PAGE.

### RNase P assay

RNase P activity assays were carried out and analysed as previously described (17,23). Substrates at 3–10 nM were cleaved with reconstituted recombinant mtRNase P at 200 nM ((mt)tRNA^Ile^) or 400 nM [(mt)tRNA^Lys^] at 30°C in the aforementioned buffer. Aliquots were withdrawn at defined intervals or at known linear-range time-points, stopped by mixing with one volume of formamide gel-loading buffer (22) and analysed by denaturing PAGE.

### Dehydrogenase assay

l-3-hydroxyacyl-CoA dehydrogenase activity was measured as acetoacetyl-CoA dependent nicotinamide adenine dinucleotide (NADH) dehydrogenation (25).

### Electrophoretic mobility shift assay

5′ end-labelled pre-tRNAs at 1 nM were incubated with protein concentrations from 10 nM to 1.2 µM in methylation/processing buffer containing 100 mM sucrose for 30 min at room temperature. N- and C-terminally tagged TRMT10C were used, and they showed indistinguishable binding properties. RNA and RNA–protein complexes were resolved by 6% (29:1) native PAGE (0.5× TBE) at 8°C. Dried gels were analysed by storage phosphor autoradiography and image analysis using ImageQuant TL 7 (GE Healthcare). Bound (shifted) versus unbound RNA was quantitated. The bound fraction was plotted against the protein concentration and curves fit by non-linear regression (‘one site specific binding with Hill slope’) using Prism 5 (GraphPad Software). RNA integrity and loss were routinely monitored by denaturing PAGE. Concentrations of TRMT10C >1 µM caused up to 50% tRNA degradation.

## RESULTS

### A subcomplex of human mtRNase P has tRNA methyltransferase activity

**Figure 2.**
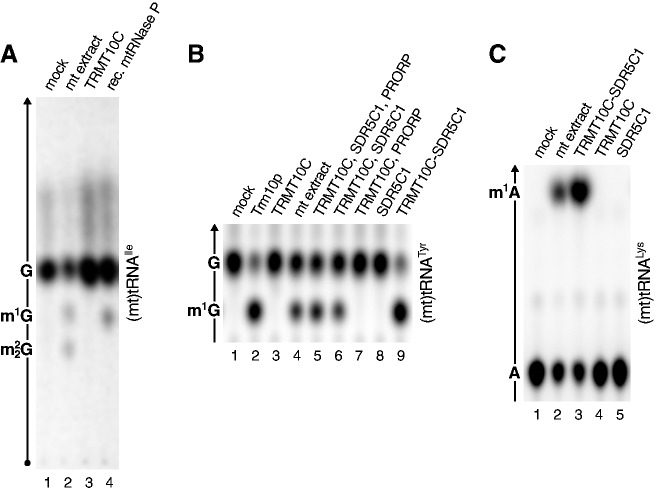
A subcomplex of human mtRNase P has tRNA methyltransferase activity. (**A**) *In vitro* transcribed (mt)tRNA^Ile^ labelled by [α-^32^P]guanosine triphosphate was incubated with SAM and either HeLa cell mitochondrial extract (mt extract; lane 2), recombinant TRMT10C (lane 3) or mtRNase P reconstituted from its recombinant components (TRMT10C, SDR5C1, PRORP; lane 4). The tRNA hydrolysate was resolved by TLC; origin and direction of migration, and the positions of guanosine monophosphate (G) and its methylated derivatives (m^1^G and 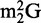) are indicated to the left. (**B**) (Mt)tRNA^Tyr^ specifically labelled at the linkage between tRNA-position 8 and 9 only was incubated with HeLa cell mitochondrial extract (lane 4), with the indicated recombinant proteins (lanes 2, 3, 8), with combinations (mixtures) of those (lanes 5–7) or with recombinant TRMT10C and SDR5C1 purified as a complex (lane 9). The tRNA hydrolysate was resolved by TLC; direction of migration and the positions of G and m^1^G are indicated to the left; only the informative part of the TLC is shown. (**C**) (Mt)tRNA^Lys^ labelled at the linkage between tRNA-position 8 and 9 was incubated with HeLa cell mitochondrial extract (lane 2), with the recombinant TRMT10C–SDR5C1 complex (lane 3) or with TRMT10C and SDR5C1 separately (lanes 4, 5). The tRNA hydrolysate was resolved by TLC; direction of migration and the positions of A and m^1^A are indicated to the left; only the informative part of the TLC is shown.

Yeast Trm10p transfers the methyl group of SAM to the *N*^1^ of G9 in tRNAs [Figure 1C (19)]. A corresponding enzymatic activity was previously found in human mitochondrial extracts (26). We investigated whether this activity is owned by TRMT10C and, thus, is also associated with mtRNase P. We used a (mt)tRNA^Ile^ (Figure 1D) with all guanosines labelled as a substrate, and crude HeLa cell mitochondrial extracts or tagged recombinant proteins purified by affinity chromatography (Supplementary Figure S1) for *in vitro* methylation assays; the RNA was subsequently isolated, hydrolysed and 5′ nucleoside monophosphates resolved by TLC (Figure 2A). Mitochondrial extract generated m^1^G and 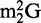 nucleosides, presumably from G9 and G26 of (mt)tRNA^Ile^ (Figure 2A, lane 2), the sites where these modifications are naturally found (Figure 1D). Surprisingly, however, TRMT10C apparently had no methyltransferase activity at all, whereas an mtRNase P reconstituted from its recombinant components produced m^1^G (Figure 2A, lanes 3 and 4). To verify that m^1^G derives from position 9, we used in the following tRNA substrates specifically labelled at the linkage between position 8 and 9, so that only 5′ nucleoside monophosphates derived from position 9 would be labelled in the hydrolysate. Like Trm10p, a mitochondrial extract and the mtRNase P holoenzyme, but not TRMT10C on its own, generated m^1^G9 (Figure 2B, lanes 2–4). We then dissected the involvement of the three individual protein components of mtRNase P (TRMT10C, SDR5C1, PRORP) in the methyltransferase reaction. TRMT10C and SDR5C1 were necessary and sufficient to reconstitute a methyltransferase activity equivalent to yeast Trm10p (Figure 2B, lanes 5–8). The identity of the modified nucleoside (m^1^G) was confirmed by 2D TLC and comparison with the Trm10p-generated methylation product and with reference maps [Supplementary Figure S2A–C (24)]. The transfer of radioactivity from *S*-adenosyl [methyl-^14^C]-methionine to an unlabelled tRNA substrate, moreover, confirmed that the methyl group is derived from SAM (Supplementary Figure S2D).

Unlike the prototypic yeast Trm10p and its archaeal homologues (16,19), TRMT10C requires another protein, SDR5C1, to be active as a methyltransferase. As an alternative to the reconstitution of the holoenzyme by mixing of purified recombinant TRMT10C and SDR5C1, we developed a protocol to purify the two proteins as an active complex. Purified His-tagged SDR5C1 (Supplementary Figure S1, lane 4) was saturated with an excess of native untagged TRMT10C (in the form of a crude bacterial lysate), and the mixture of the two proteins was subsequently subjected to affinity chromatography. The purified complex (Supplementary Figure S1, lane 5) was apparently stable and active as a methyltransferase (Figure 2B, lane 9).

Only 5 of the 22 (mt)tRNAs have a guanosine at position 9, whereas in 14 cases an adenosine is found. G9 and A9 are apparently *N*^1^-methylated in the mature (mt)tRNAs of animals (15). A tRNA:m^1^A9 methyltransferase activity was previously demonstrated in human mitochondrial extracts (26). Although modification enzymes are generally highly specific with respect to the kind of modification introduced, the TRMT10C–SDR5C1 complex was nevertheless an obvious candidate to be responsible for tRNA:m^1^A9, in addition to tRNA:m^1^G9 formation. Indeed, m^1^A9 was generated from the A9-containing (mt)tRNA^Lys^ by mitochondrial extract or TRMT10C–SDR5C1, but again not by TRMT10C or SDR5C1 alone (Figure 2C). The identity of m^1^A was confirmed by 2D TLC and by alkali induced isomerization to m^6^A [Dimroth rearrangement (27); Supplementary Figure S2E and F]. Thus, the TRMT10C–SDR5C1 subcomplex of human mtRNase P is a bifunctional tRNA:m^1^R9 methyltransferase, that is, it is responsible for the formation of m^1^G9 and m^1^A9 in human (mt)tRNAs.

### TRMT10C and SDR5C1 constitute the tRNA:m^1^R9 methyltransferase *in vivo*

**Figure 3.**
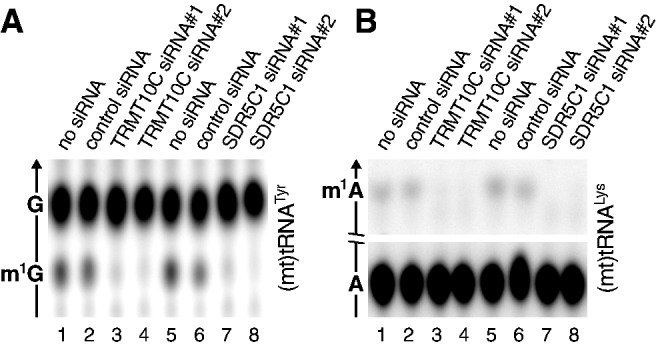
Loss of tRNA:m^1^G9 and tRNA:m^1^A9 methyltransferase activity in HeLa cell mitochondrial extracts after RNAi-mediated knock-down of TRMT10C or SDR5C1 mRNA. Cells were transfected with a control siRNA or with two different gene specific siRNAs, respectively. Forty-eight hours after transfection, total RNA was extracted from a sample, and relative TRMT10C and SDR5C1 mRNA levels were determined by quantitative real-time RT-PCR and were normalized to glyceraldehyde-3-phosphate dehydrogenase (GAPDH) (control siRNA, 116%; TRMT10C siRNA#1, 33%; TRMT10C siRNA#2, 18%; control siRNA, 119%; SDR5C1 siRNA#1, 14%; SDR5C1 siRNA#2, 4%). Based on previously established knock-down kinetics, mitochondrial extracts were prepared 3 days after transfection in the TRMT10C RNAi experiment and 6 days after transfection in the SDR5C1 RNAi experiment (17). Extracts were adjusted for their protein concentration. (**A**) tRNA:m^1^G9 methyltransferase activity in mitochondrial extracts of siRNA-treated HeLa cells. Position 9-labelled (mt)tRNA^Tyr^ was used as substrate, and the tRNA hydrolysate was resolved by TLC. (**B**) tRNA:m^1^A9 methyltransferase activity in mitochondrial extracts of siRNA-treated HeLa cells. Position 9-labelled (mt)tRNA^Lys^ was used as substrate. The middle part of the TLC was cropped as indicated and only the informative part is shown.

To test whether TRMT10C and SDR5C1 are responsible for the mitochondrial tRNA:m^1^R9 methyltransferase activity *in vivo*, their genes were silenced in HeLa cells by RNAi. We used siRNAs previously used to characterize human mtRNase P (17) and achieved a comparable reduction of TRMT10C and SDR5C1 mRNA levels (see legend to Figure 3). Compared with control samples, the tRNA:m^1^G9 and tRNA:m^1^A9 methyltransferase activity of mitochondrial extracts was largely eliminated by the knock-down of either TRMT10C or SDR5C1 (Figure 3A and B). Consistent with these results, others recently observed a reduction of the typical sequence misreads at position 9 of (mt)tRNAs on *TRMT10C* silencing [m^1^G and m^1^A result in sequencing error at modified sites (28,29)]. Altogether, results obtained *in vitro* and *in vivo* demonstrate that TRMT10C and SDR5C1 are essential for R9 methylation in human mitochondria, and that there is no major backup pathway to modify position 9 in (mt)tRNAs.

### Methyltransferase and mtRNase P are functionally independent

**Figure 4.**
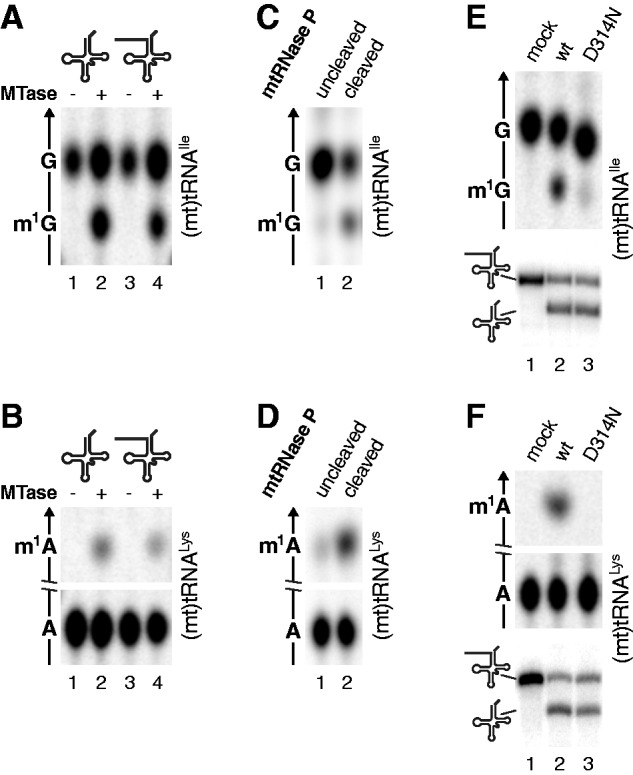
The methyltransferase activity of the TRMT10C–SDR5C1 complex and its contribution to mtRNase P activity are functionally independent. (**A** and **B**) A 5′ extension does not interfere with tRNA methylation. Position 9-labelled tRNAs with mature 5′ end (lane 2) or with 5′ extension (lane 4) were methylated with the TRMT10C–SDR5C1 methyltransferase (MTase) complex and the RNA hydrolysates resolved by TLC; (A) (mt)tRNA^Ile^; (B) (mt)tRNA^Lys^. (**C** and **D**) Methylation and cleavage are not coupled. tRNA substrates with 5′ extension were partially (∼30%) cleaved with reconstituted mtRNase P (TRMT10C–SDR5C1, PRORP) in the presence of SAM. Uncleaved and cleaved tRNA were separated by PAGE, isolated and analysed for the presence of a methyl group at position 9 by hydrolysis and TLC; (C) (mt)tRNA^Ile^; (D) (mt)tRNA^Lys^. (**E** and **F**) The functional integrity of the methyltransferase is not required to support cleavage by mtRNase P. tRNA substrates with 5′ extension were incubated with wild-type (wt) or mutant (D314N) TRMT10C-containing methyltransferase (TRMT10C–SDR5C1; upper panels) or reconstituted mtRNase P (TRMT10C–SDR5C1, PRORP; lower panels), and the reaction products separated by TLC (upper panels) or denaturing PAGE (lower panels; substrate and cleavage product indicated to the left); (E) (mt)tRNA^Ile^; (F) (mt)tRNA^Lys^.

In view of the association of the methyltransferase and 5′-processing activity within mtRNase P, we investigated a possible functional interrelation of the two enzymatic activities. To assess whether the removal of the 5′ leader sequence is a prerequisite for a tRNA to be methylated by the TRMT10C–SDR5C1 (sub)complex, we used tRNA molecules with or without 5′ extension in methyltransferase assays. A 5′ leader sequence did not prevent the enzyme from transfering the methyl group to G9 or A9 of the tRNA (Figure 4A and B). Thus, precursors and mature tRNAs are substrates for the mitochondrial tRNA:m^1^R9 methyltransferase complex.

Although the catalytic site for tRNA cleavage is reasonably supposed to reside in PRORP, also TRMT10C and SDR5C1 are essential for RNase P activity *in vitro* and *in vivo* (17,18). Removal of the 5′ leader *in vitro* does not require a methyl group donor (17,23); thus, there is no need for tRNA methylation to precede cleavage. Nevertheless, it was intriguing to conjecture an actual coupling of the two reactions in the multifunctional complex. To test this hypothesis, a 5′-extended tRNA substrate was partially (∼30%) cleaved *in vitro* by incubation with an excess of mtRNase P holoenzyme in the presence of SAM. Uncleaved and cleaved RNA were separated by PAGE, isolated and were subsequently analysed for the presence of a methyl group at tRNA-position 9. The cleaved tRNAs, although productively contacted by the mtRNase P complex for processing, were only partially methylated (Figure 4C and D). Thus, even if allowed to proceed in association, the two reactions were not coupled. The incomplete methylation of the cleavage product, despite its obvious contact with the methyltransferase, is consistent with a slower kinetics of tRNA methylation compared with 5′-end cleavage. Methylation of the uncleaved 5′-extended fraction confirms that such tRNAs are substrate for the methyltransferase activity.

To test whether the functional integrity of the methyltransferase is required to sustain efficient tRNA precursor (pre-tRNA) processing by the mtRNase P holoenzyme, we generated a mutant variant of TRMT10C, in which the aspartate residue 314 was replaced by asparagine (D314N). D314 of TRMT10C is an invariant amino acid conserved throughout the TRM10 family and was proposed to be part of these enzymes’ active site (16). Substitution D314N indeed impaired the methyltransferase activity of the TRMT10C–SDR5C1 complex on A9 and G9 containing substrates (Figure 4E and F). The pre-tRNA processing activity of an mtRNase P holoenzyme reconstituted with the D314N-mutant TRMT10C, nevertheless, was unaffected in its ability to remove the 5′ leader sequence from the same substrates (Figure 4E and F). These experiments provide first experimental evidence for an aspartate residue apparently being part of the methyltransferase’s active site and demonstrate that the different roles of TRMT10C are functionally independent from each other.

### All three human TRM10 homologues have tRNA methyltransferase activity

Besides TRMT10C, vertebrate genomes encode two further homologues of yeast TRM10, TRMT10A and TRMT10B. These two proteins do not carry a mitochondrial targeting signal and have higher sequence homology to TRM10 than to TRMT10C, and by these criteria, they seem to represent the true orthologues of TRM10, whereas TRMT10C represents a paralogue with altered subcellular localization and added functionality as an RNase P subunit. Still, none of the animal TRM10 homologues has been tested for its methyltransferase activity to date.

**Figure 5.**
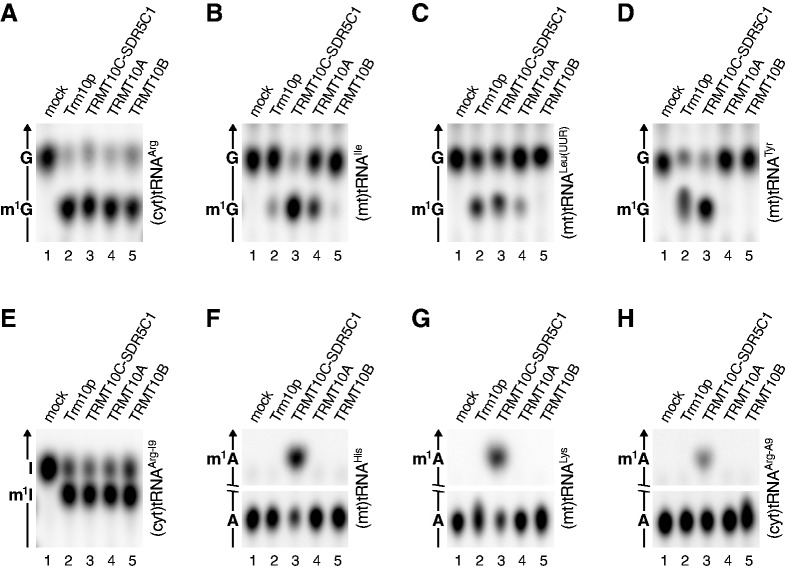
All three human TRM10 homologues have methyltransferase activity, but differ in their substrate specificity. Recombinant yeast Trm10p, the human TRMT10C–SDR5C1 complex and human TRMT10A and TRMT10B were assayed with different human tRNA substrates labelled at position 9, and the tRNA hydrolysates were resolved by TLC. (**A**) (Cyt)tRNA^Arg^; (**B**) (mt)tRNA^Ile^; (**C**) (mt)tRNA^Leu(UUR)^; (**D**) (mt)tRNA^Tyr^; (**E**) (cyt)tRNA^Arg-I9^ (G9 replaced by inosine); (**F**) (mt)tRNA^His^; (**G**) (mt)tRNA^Lys^; (**H**) (cyt)tRNA^Arg-A9^ (G9 replaced by adenosine).

We investigated the enzymatic activity of human TRMT10A and TRMT10B and compared the two putative methyltransferases to Trm10p and the TRMT10C–SDR5C1 complex. We tested a cytosolic tRNA, (cyt)tRNA^Arg^, which contains m^1^G9 and should thereby represent a possible natural substrate of TRMT10A and/or TRMT10B (15). Indeed, TRMT10A and TRMT10B efficiently methylated human (cyt)tRNA^Arg^ (Figure 5A). However, when we tested three G9-containing (mt)tRNAs, only some of them were methylated by TRMT10A and TRMT10B, suggesting a partial incompatibility with the non-canonically structured (mt)tRNAs (Figure 5B–D). All the G9-containing tRNAs tested were efficiently methylated by the mitochondrial TRMT10C–SDR5C1 complex, yet also by Trm10p, an enzyme naturally acting on canonical cytosolic tRNAs.

The tRNA:m^1^G37 methyltransferase TRM5 methylates inosine in addition to guanosine if inosine is present at tRNA position 37 as a product of adenosine deamination (30,31). Notably, like guanosine, but in contrast to adenosine, inosine’s *N*^1^ is protonated at physiological pH (Figure 1B and D; Supplementary Figure S3A). Although inosine is not found in tRNAs at position 9, we were curious whether an artificially introduced I9 would be converted to m^1^I9 by the studied TRM10 homologues. A derivative of human (cyt)tRNA^Arg^, in which G9 had been changed to I9, was efficiently methylated by Trm10p and all its three human homologues (Figure 5E; we confirmed the identity of m^1^I by 2D TLC; Supplementary Figure S3B). Thus, m^1^G methyltransferases generally seem to be able to methylate inosine too.

In eukarya, m^1^A9 was so far only found in animal (mt)tRNAs (15). Therefore, Trm10p, TRMT10A and TRMT10B would not reasonably be supposed to have tRNA:m^1^A9 methyltransferase activity. Indeed, when we tested two A9-containing (mt)tRNAs or an A9-derivative of (cyt)tRNA^Arg^ in methylation assays with the different TRM10 homologues, only the mitochondrial TRMT10C–SDR5C1 complex generated m^1^A9 (Figure 5F–H). Thus, the mitochondrial TRMT10C–SDR5C1 complex is the only dual specific tRNA:m^1^R9 methyltransferase among the examined eukaryal TRM10 homologues, methylating any purine at tRNA-position 9, apparently irrespective of its identity or context.

### The dehydrogenase function of SDR5C1 is dispensable for methylation and cleavage

**Figure 6.**
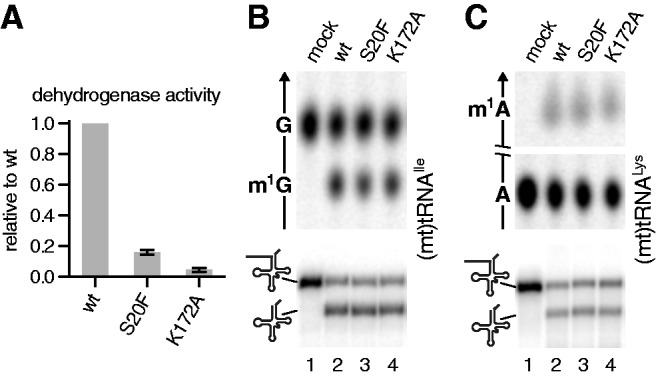
The dehydrogenase function of SDR5C1 is not required for tRNA methylation or cleavage. Amino acid substitutions were introduced into the cofactor-binding site (S20F), and the active site (K172A) of SDR5C1 and recombinant TRMT10C–SDR5C1 complexes was purified. (**A**) Relative l-3-hydroxyacyl-CoA dehydrogenase activity of SDR5C1 mutants determined with acetoacetyl-CoA as substrate; mean and standard deviation of triplicates. (**B** and **C**) Methyltransferase (upper panels) and mtRNase P (lower panels) activity of recombinant enzyme preparations with mutant SDR5C1 subunits; (B) (mt)tRNA^Ile^ with 5′ extension; (C) (mt)tRNA^Lys^ with 5′ extension.

In contrast to all other members of the TRM10 family characterized so far, TRMT10C requires a partner protein, SDR5C1. The role of SDR5C1 in tRNA processing has remained obscure since its identification as a component of mtRNase P (17). It is a member of the ubiquitous short-chain dehydrogenase/reductase family and uses NAD(H) as cofactor (32,33). Conserved sequence elements of this family are a classical Rossmann-fold nucleotide cofactor binding site and an active site with an Asn-Ser-Tyr-Lys tetrad. We sought to explore the relationship between the dehydrogenase moiety and the tRNA maturation activities of the mtRNase P, with the help of SDR5C1 point mutants. Single amino acid substitutions were introduced in the cofactor-binding site (S20F) and in the catalytic tetrad (K172A) by site-directed mutagenesis. In a dehydrogenase assay, the catalytic activity of the S20F mutant was reduced to 16% in comparison with the wild-type, whereas the activity of the K172A mutant was completely abolished (Figure 6A). Nevertheless, when the same mutant proteins were used to reconstitute the methyltransferase subcomplex or mtRNase P, none of these enzymatic activities was impaired (Figure 6B and C). These results show that not only the dehydrogenase activity of SDR5C1 is dispensable for tRNA methylation or 5′ leader removal but also the functional integrity of the NAD(H) binding site is not required.

### TRMT10C is responsible for catalysis and substrate recognition

**Figure 7.**
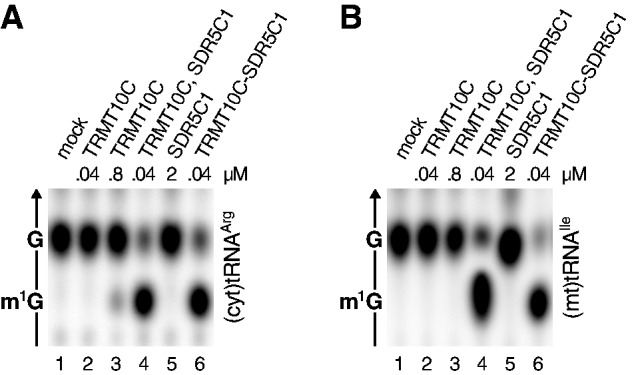
TRMT10C has vestigial methyltransferase activity. TRMT10C was tested for tRNA:m^1^G9 methyltransferase activity at low (40 nM; lane 2) and high concentration (800 nM; lane 3), and at low concentration in the presence of an excess (2 µM) of SDR5C1 (lane 4) or purified as TRMT10C–SDR5C1 complex (lane 6) with different tRNA substrates; (**A**) (cyt)tRNA^Arg^; (**B**) (mt)tRNA^Ile^.

A structural comparison of TRMT10C to its paralogues did not reveal any obvious clue for the requirement of an additional subunit. TRMT10C still seems to be the enzyme’s catalytic subunit, and we sought to determine whether it might have some residual methyltransferase activity in the absence of SDR5C1 under certain conditions. We made use of human cytosolic tRNA^Arg^, which was most efficiently methylated *in vitro* by all tested enzymes (Figure 5A). A weak m^1^G spot was detectable after prolonged incubation with high amounts of TRMT10C alone (Figure 7A, lane 3). Under the same conditions, a 20-fold lower amount of TRMT10C accomplished substrate methylation to near completeness if supplemented with an excess of SDR5C1 or pre-associated with it (Figure 7A, lanes 4 and 6). (Mt)tRNA^Ile^, however, was not methylated even by high amounts of TRMT10C alone (Figure 7B), thereby confirming previous findings (Figure 2). TRMT10C, thus, contains a vestigial methyltransferase active site, but it needs to associate with SDR5C1 for efficient catalysis.

**Figure 8.**
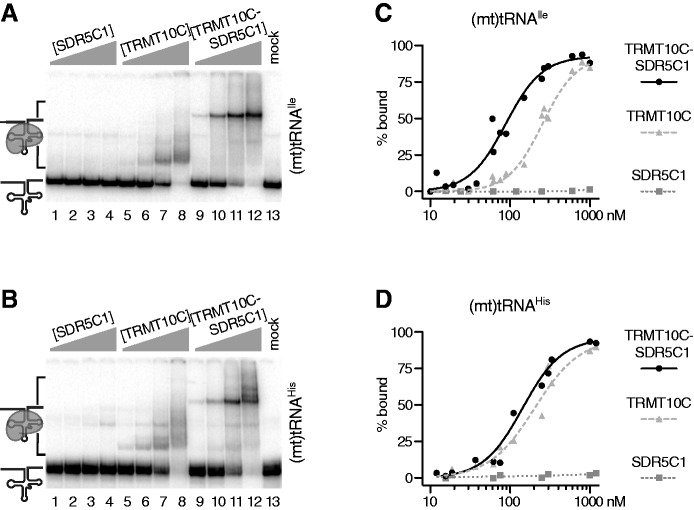
TRMT10C is the subunit primarily responsible for tRNA binding. (**A** and **B**) Labelled pre-tRNAs were incubated with increasing concentrations of SDR5C1, TRMT10C or the TRMT10C–SDR5C1 complex and were resolved by native PAGE; representative gels are shown. The concentration of the TRMT10C–SDR5C1 complex refers to its TRMT10C content. (**C** and **D**) Binding data from independent experiments were plotted as single data points against protein concentration and curves fit by non-linear regression. (A and C) (Mt)tRNA^Ile^; (B and D) (mt)tRNA^His^.

We had previously hypothesized that SDR5C1 could contribute to RNA binding in the mtRNase P holoenzyme (17,34) and consequently the methyltransferase subcomplex as well. To investigate whether SDR5C1 is indeed directly involved in the interaction of enzyme and RNA substrate, we compared the tRNA binding properties of the TRMT10C–SDR5C1 complex with those of its individual components. In an electrophoretic mobility shift assay, the TRMT10C–SDR5C1 complex retarded the migration of (mt)pre-tRNA^Ile^ and (mt)pre-tRNA^His^ in a concentration dependent manner (Figure 8A and B, lanes 9–12), and we derived dissociation constants (*K*_D_) of 88 ± 8 and 143 ± 21 nM, respectively (Figure 8C and D). Similarly, TRMT10C was binding to both pre-tRNAs, yet, it was causing a smaller shift than the TRMT10C–SDR5C1-tRNA complex, consistent with its lower molecular weight (Figure 8A and B, lanes 5–8). Compared with the TRMT10C–SDR5C1 complex, the pre-tRNA-binding affinity of TRMT10C alone was slightly lower, with an apparent *K*_D_ of 255 ± 10 nM for (mt)tRNA^Ile^ and 203 ± 15 nM for (mt)tRNA^His^ (Figure 8C and D). SDR5C1 alone, however, did not bind to RNA even at micromolar concentration (Figure 8). These results suggest that SDR5C1 has no direct role in substrate recognition by the TRMT10C–SDR5C1 complex, and TRMT10C is the subunit primarily interacting with the tRNA.

## DISCUSSION

The widespread, possibly ubiquitous, *N*^1^-methylation of purines in position 9 of animal (mt)tRNAs suggests a critical importance of this modification for (mt)tRNA function. Here, we showed that a subcomplex of human mtRNase P has the required tRNA:m^1^G9 and tRNA:m^1^A9 methyltransferase activities and is the enzyme responsible for the methylation of (mt)tRNAs. The unprecedented and unanticipated nature of this methyltransferase, made up of two subunits, had apparently prevented the previous identification and characterization of this crucial mitochondrial enzyme. The integration of this bifunctional methyltransferase complex into mtRNase P, moreover, demonstrates a perplexing extent of moonlighting among mitochondrial proteins and indicates a level of organizational complexity so far not observed in tRNA maturation systems.

In the (cyt)tRNAs of Eukarya, A9 is not modified, and the methylation of G9 to m^1^G9 is restricted to a subset of (cyt)tRNAs (15). In yeast, Trm10p, the eponymous member of the TRM10 family, is the sole tRNA:m^1^G9 methyltransferase, yet its function is not essential under standard growth conditions, pointing to a less stringent requirement of this modification in (cyt)tRNAs (19). Here, we showed that the two non-mitochondrial human TRM10 homologues TRMT10A and TRMT10B have tRNA:m^1^G9 methyltransferase activity like yeast Trm10p and, thus, likely represent its orthologues. It is not known why vertebrates have two tRNA:m^1^G9 methyltransferases for their (cyt)tRNAs, whereas one is sufficient in all other eukarya; the two might have different developmental or tissue-specific functions or have distinct subcellular distribution or substrate specificities. Interestingly, Trm10p seems to be more promiscuous with respect to substrate selectivity than TRMT10A and TRMT10B, methylating all tested G9-containing substrates (Figure 5), although *in vivo* it also selectively methylates only a specific set of (cyt)tRNAs. A more systematic study of the selectivity of cytosolic tRNA:m^1^G9 methyltransferases will be required to uncover the molecular basis of their substrate discrimination.

Animals are apparently the only eukaryal group encoding more than one *TRM10* homolog, that is, generally two and even three in vertebrates, and the animal homologue with the higher degree of sequence divergence from Trm10p typically carries an N-terminal mitochondrial targeting sequence (17,19). Consistently, methylation of tRNA position 9 is confined to animal mitochondria, whereas the (mt)tRNAs of all other eukaryal groups have remained bacterial-like with respect to the lack of this modification and tRNA structure (15). It seems plausible that a form of TRM10 and the associated methylation of G9 were recruited to mitochondria and extended to R9 during evolution to stabilize the fold of the otherwise unstable non-canonical animal (mt)tRNAs. The ability of the human mitochondrial enzyme to methylate guanosine and adenosine is unusual among tRNA modification enzymes, but not entirely unprecedented. A homologue of TRM10 in the Archaeon *Thermococcus kodakaraensis* was recently reported to be able to methylate A9 and G9 in tRNAs (16). Moreover, the same authors observed that the TRM10 homologue of *Sulfolobus acidocaldarius* is able to methylate A9 but not G9, illustrating the flexibility of the TRM10 family to evolve towards different purine specificities.

Based on structural features, the TRM10 family belongs to the SpoU-TrmD (SPOUT) superfamily, a large class of methyltransferases characterized by an α/β knot fold (35). However, TRM10 is the most diverged among the SPOUT methyltransferases, and no crystal structure is available. The superfamily member functionally most closely related to TRM10 is bacterial TrmD, as it catalyses the methylation of *N*^1^ of G37 in tRNAs (36) and may serve as a paradigm for the potential catalytic mechanism of m^1^G formation. In the active site of TrmD, an aspartate is proposed to act as a general base to deprotonate *N*^1^ of guanosine, whereas an arginine stabilizes the consequent *O*^6^ negative charge and a glutamate interacts with *N*^2^; the activated *N*^1^ may then make a direct nucleophilic attack on the reactive methyl group of SAM (37). m^1^A, the other reaction product of the mitochondrial tRNA:m^1^R9 methyltransferase, is also found at tRNA positions 14, 22 and 58 (15), and the best characterized enzyme is the one responsible for m^1^A58 in Archaea, the two-subunit enzyme TrmI (38). At physiological pH, *N*^1^ of adenosine is not protonated, and an aspartate deprotonates the exocyclic *N*^6^, inducing the formation of the imino tautomer that activates the lone electron pair of *N*^1^ for attack on the methyl group of SAM. TrmD and TrmI thereby exemplify the different mechanisms required for m^1^G and m^1^A formation. Without any information about the active site geometry of TRM10 methyltransferases, it seems currently impossible to understand how one catalytic domain achieves the methylation of the two differentially protonated purines or why different TRM10 family members have different base specificities.

TRMT10Cs dependence on another subunit for methyltransferase activity is unique within the TRM10 family. The second subunit of the mitochondrial tRNA:m^1^R9 methyltransferase, SDR5C1, belongs to the large family of short-chain dehydrogenases/reductases (32,33). It was reported to act as a dehydrogenase on a wide range of substrates, such as fatty acids, alcohols and hydroxysteroids, and it was found to be essential for isoleucine degradation and short branched-chain fatty acid β-oxidation *in vivo* (39). None of these dehydrogenase activities has a direct connection with RNA metabolism, and we, moreover, showed that the integrity of SDR5C1s active site is not essential for its contribution to methyltransferase and RNase P activity (see K172A mutant in Figure 6). SDR5C1 also has a typical Rossmann-fold NAD(H) dinucleotide-binding domain; this kind of domain was shown to mediate the binding of RNA by a number of metabolic enzymes and was proposed to be a common RNA-binding motif (40,41). However, we did not observe any RNA binding by SDR5C1 alone, and it only slightly increased the tRNA affinity of TRMT10C in our electrophoretic mobility shift experiments (Figure 8). Likewise, the bulky phenylalanine residue in the SDR5C1–S20F mutant blocked the dinucleotide-binding pocket to an extent that almost abolished its dehydrogenase activity, but did not affect the methyltransferase or RNase P activity of the reconstituted complex (Figure 6). Consistently, an excess of NAD^+^ or NADH did not inhibit the tRNA:m^1^R9 methyltransferase or mtRNase P (data not shown). Thus, SDR5C1 has no direct role in tRNA binding. Moreover, all methyltransferase functions seem to reside in TRMT10C, based on its homology to other TRM10 family members and its vestigial catalytic activity (Figure 7). The involvement of SDR5C1 in the methyltransferase complex remains enigmatic, and its essential contribution to the methyltransferase complex might in the end be a mere structural stabilization or remoulding of the actual catalytic component TRMT10C. Structural studies will be required to shed light on the dual-specific methyltransferase mechanism, the relationship between TRMT10C and SDR5C1 and on the actual role of the two subunits in tRNA methylation and 5′-end processing.

SDR5C1 behaves like a prototypical moonlighting enzyme (42), a multifunctional protein performing multiple unrelated functions independent from each other—fatty acid β-oxidation and tRNA methylation. In turn, the TRM10C–SDR5C1 methyltransferase complex moonlights in 5′ processing of pre-tRNAs, adding a further level of complexity. Human mtRNase P, thus, constitutes a multifunctional complex, gathering different enzymatic activities involved in tRNA maturation. Homologues of PRORP, the nucleolytic subunit of human mtRNase P, were recently shown to be active on their own (43–45). The requirement of human PRORP for additional proteins is a peculiarity of mtRNAse P that is strikingly reminiscent of TRMT10C, which, unlike any other TRM10 family member, requires a partner protein. Moreover, the physical association of different functions, although not mirrored by a strict functional coupling, might suggest the existence of a higher-order association of related functions in human mitochondria. It is intriguing to speculate that more enzymes might be in association with human mtRNase P *in vivo*, constituting a sort of mitochondrial ‘RNA factory’, optimizing the handing of immature mitochondrial RNAs through the series of steps required for their maturation.

## SUPPLEMENTARY DATA

Supplementary Data are available at NAR Online: Supplementary Table 1, Supplementary Figures 1–3 and Supplementary Reference [46].

## FUNDING

Vienna Science and Technology Fund (WWTF) [LS09-032]; Austrian Science Fund (FWF) [P17453]. Funding for open access charge: Vienna Science and Technology Fund (WWTF) [LS09-032].

*Conflict of interest statement*. None declared.

## Supplementary Material

Supplementary Data
