# A subcomplex of human mitochondrial RNase P is a bifunctional methyltransferase – extensive moonlighting in mitochondrial tRNA biogenesis

**DOI:** 10.1093/nar/gky931

**Published:** 2018-10-08

**Authors:** Elisa Vilardo, Christa Nachbagauer, Aurélie Buzet, Andreas Taschner, Johann Holzmann, Walter Rossmanith

*Nucl. Acids Res. 40 (22): 11583–11593. doi: 10.1093/nar/gks910*

The authors wish to draw attention to an error in their published article.

In 2012, we reported that all three human TRM10 homologues have tRNA methyltransferase activity. However, in a recent follow-up study, we were not able to reproduce the reported tRNA:m^1^G9 methyltransferase activity of human TRMT10B with fresh enzyme preparations. This prompted us to revisit the original preparation used in 2012, and mass spectrometry analysis of the concerned TRMT10B preparation revealed a significant contamination with TRMT10A, suggesting that the tRNA:m^1^G9 methyltransferase activity previously observed was in fact due to TRMT10A rather than TRMT10B. The contamination was not previously noticed, because the two recombinant proteins have a similar molecular weight and were therefore not resolved by SDS-PAGE.

We now repeated the complete set of experiments shown in Figure 5 of the 2012 article and a new, revised Figure 5 is provided below. In contrast to the previously reported results, now none of the 8 tested tRNA substrates was methylated by TRMT10B. Thus, it remains unclear whether TRMT10B has a more restricted substrate specificity than the other studied TRM10 homologues, has at all tRNA methyltransferase activity, or, like TRMT10C, requires (a) cofactor(s) for activity. The use of fresh enzyme preparations, all at a final concentration of 250 nM (i.e. in far excess over the substrate), moreover now showed that Trm10p, TRMT10A and TRMT10C-SDR5C1 in fact are rather promiscuous tRNA:m^1^G9 methyltransferases that were able to methylate all the tested cytosolic and mitochondrial tRNAs (Figure 5A–D). All three enzymes, but not TRMT10B, were also able to methylate an artificial, inosine-containing substrate to m^1^I9 (Figure 5E), but still only the mitochondrial TRMT10C-SDR5C1 complex methylated A9 in the two tested (mt)tRNAs and the A9-derivative of (cyt)tRNA^Arg^ (Figure 5F–H).

**Figure 5.**
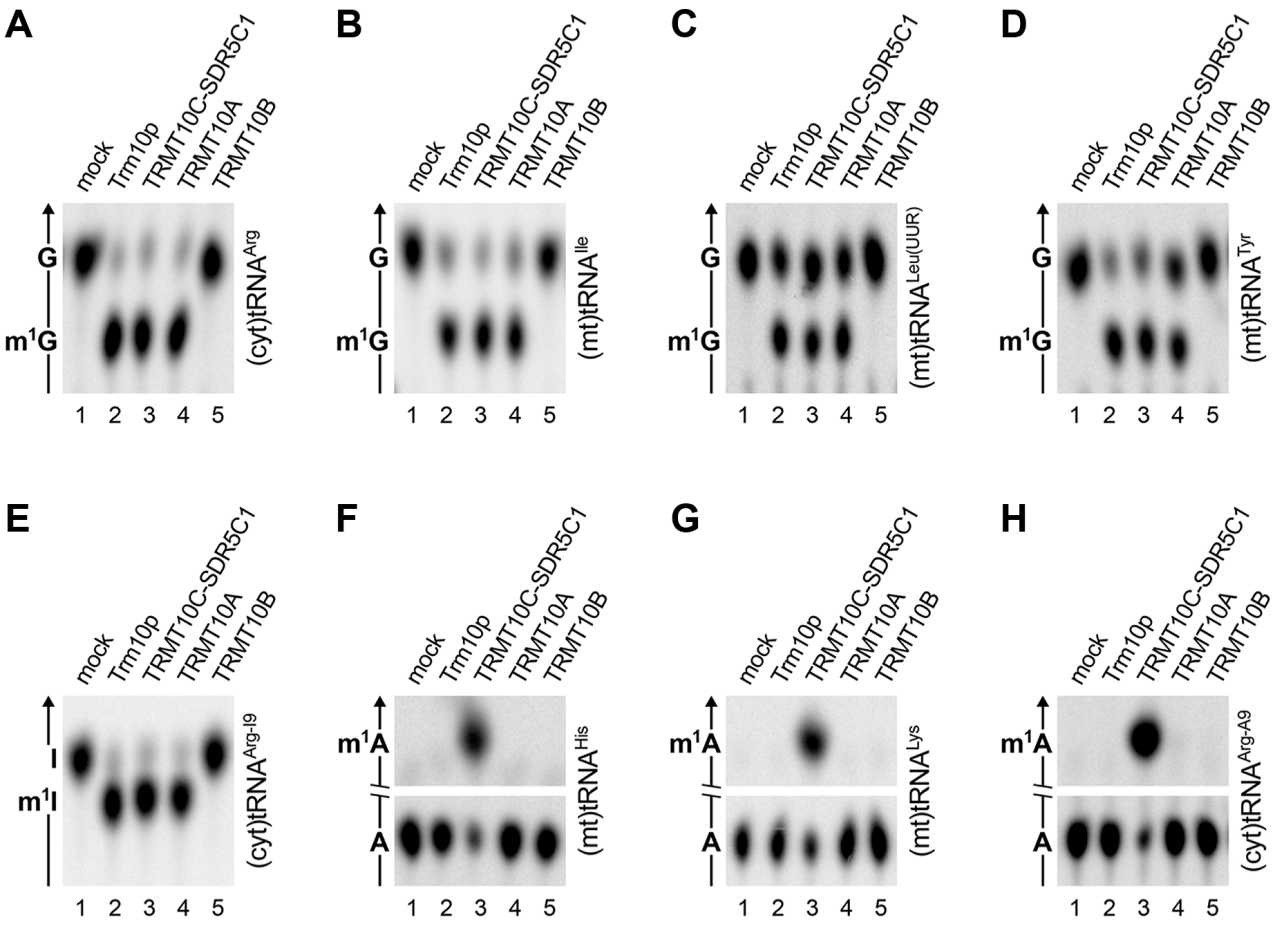
A comparison of the methyltransferase activities of yeast Trm10p and the three human TRM10 homologs. Recombinant yeast Trm10p, the human TRMT10C-SDR5C1 complex, and human TRMT10A and TRMT10B were assayed with different human tRNA substrates labelled at position 9, and the tRNA hydrolysates were resolved by TLC. (**A**) (Cyt)tRNA^Arg^; (**B**) (mt)tRNA^Ile^; (**C**) (mt)tRNA^Leu(UUR)^; (**D**) (mt)tRNA^Tyr^; (**E**) (cyt)tRNA^Arg-I9^ (G9 replaced by inosine); (**F**) (mt)tRNA^His^; (**G**) (mt)tRNA^Lys^; (**H**) (cyt)tRNA^Arg-A9^ (G9 replaced by adenosine).

All other findings and conclusion of the article are not affected and remain valid.

The authors apologise to the readers for this error and any inconvenience caused.

