# Supplementary material for: A subcomplex of human mitochondrial RNase P is a bifunctional methyltransferase—extensive moonlighting in mitochondrial tRNA biogenesis
Source: Nucleic Acids Res. 2012 Oct 5;40(22):11583–93. doi: 10.1093/nar/gks910 (PMC3526285; doi:10.1093/nar/gks910)
Supplement: Supplementary Data [file supp_40_22_11583__index.html]

A subcomplex of human mitochondrial RNase P is a bifunctional methyltransferase—extensive moonlighting in mitochondrial tRNA biogenesis — A subcomplex of human mitochondrial RNase P is a bifunctional methyltransferase—extensive moonlighting in mitochondrial tRNA biogenesis — Supplementary Data 

# A subcomplex of human mitochondrial RNase P is a bifunctional methyltransferase—extensive moonlighting in mitochondrial tRNA biogenesis

## Supplementary Data

files

**Files in this Data Supplement:**

- Supplementary Data - pdf file
