# Supplementary material for: A subcomplex of human mitochondrial RNase P is a bifunctional methyltransferase—extensive moonlighting in mitochondrial tRNA biogenesis
Source: Nucleic Acids Res. 2012 Oct 5;40(22):11583–93. doi: 10.1093/nar/gks910 (PMC3526285; doi:10.1093/nar/gks910)
Supplement: Supplementary Data [file supp_gks910_nar-02050-r-2012-File010.pdf]

SUPPLEMENTARY DATA

A subcomplex of human mitochondrial RNase P is a bifunctional methyltransferase – extensive moonlighting in mitochondrial tRNA biogenesis

Elisa Vilardo, Christa Nachbagauer, Aurélie Buzet, Andreas Taschner, Johann Holzmann, Walter Rossmanith*

Center for Anatomy & Cell Biology, Medical University of Vienna, 1090 Vienna, Austria

*To whom correspondence should be addressed. Tel: +43 1 40160 37512; Fax: +43 1 40160 937500; Email: walter.rossmanith@meduniwien.ac.at

SUPPLEMENTARY TABLE AND FIGURES

Supplementary Table S1 Nomenclature and synonyms of the studied proteins

	species	UniProtKB[a]	gene[b]	gene ID[c]	synonyms[d]
TRMT10C[e]	*H. sapiens*	Q7L0Y3	*TRMT10C*	54931	MRPP1, RG9MTD1
SDR5C1[f]	*H. sapiens*	Q99714	*HSD17B10*	3028	MRPP2, HADH2, ABAD, HSD10, HCD2
PRORP[g]	*H. sapiens*	O15091	*KIAA0391*	9692	MRPP3
Trm10p	*S. cerevisiae*	Q12400	*TRM10*	854060	YOL093W
TRMT10A[e]	*H. sapiens*	Q8TBZ6	*TRMT10A*	93587	RG9MTD2
TRMT10B[e]	*H. sapiens*	Q6PF06	*TRMT10B*	158234	RG9MTD3

The proteins studied in this work are listed together with their identifiers, gene symbol, and common synonyms.

[a]UniProtKB identifier.

[b]Gene symbol according to the HUGO Gene Nomenclature Committee and the *Saccharomyces* Genome Database, respectively.

[c]NCBI Entrez gene identifier.

[d]Only the more commonly used synonyms are listed. In the case of yeast TRM10 the locus tag is listed.

[e]Newly approved nomenclature reflecting the methyltransferase function of three human TRM10 homolgs and conforming to the nomenclature of other human tRNA methyltransferases.

[f]According to the recently proposed systematic nomenclature of the SDR superfamily (46).

[g]Recently proposed, common symbol/name (proteinaceous RNase P) for the gene family (18,43).

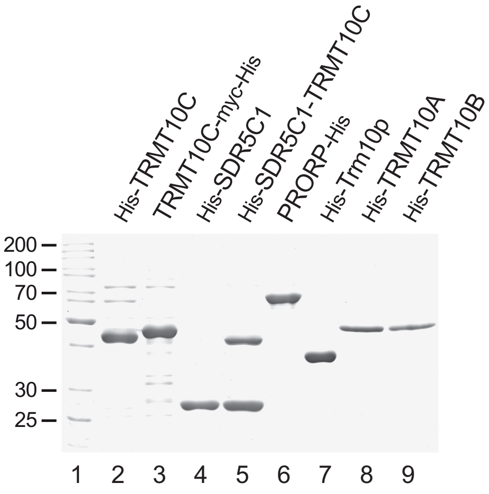

Supplementary Figure S1 Recombinant proteins used in this study. ~5 µg of the purified recombinant proteins (2.5 µg of TRMT10A and TRMT10B) were resolved by SDS-PAGE and stained with Coomassie brilliant blue. N-terminal tags precede the protein name and C-terminal tags follow the name. The molecular weight of selected marker proteins (lane 1) is indicated to the left.

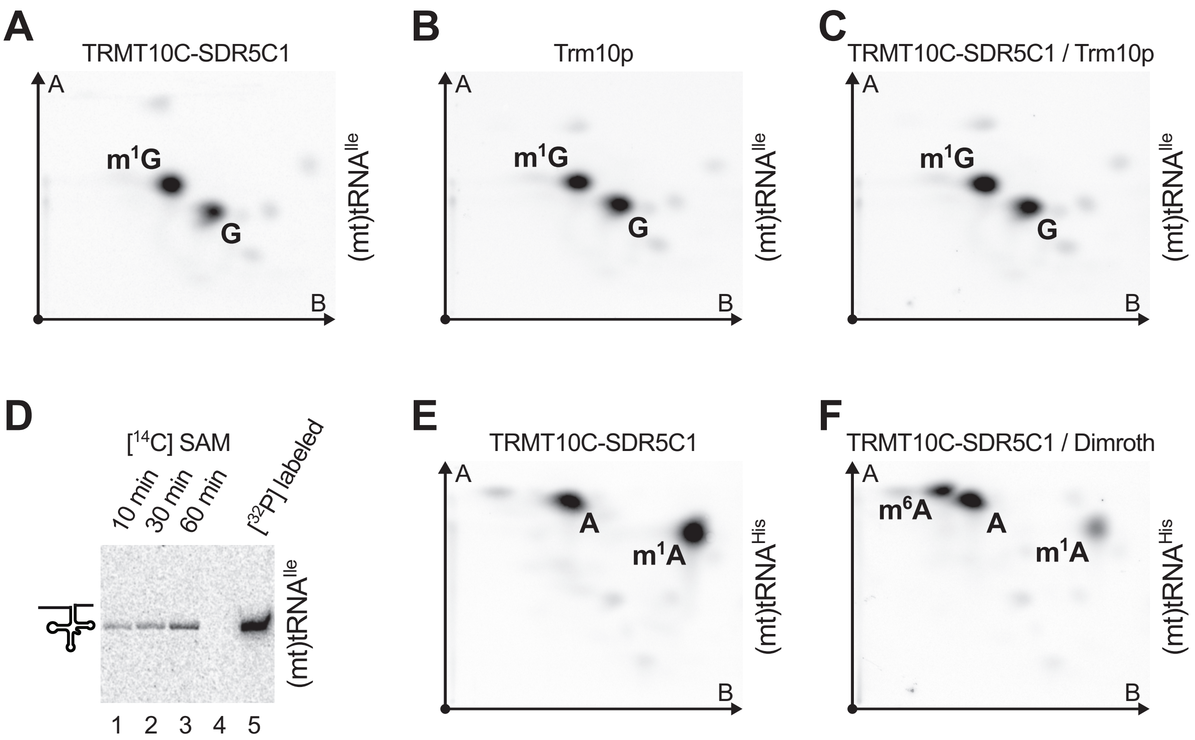

Supplementary Figure S2 Characterization of the methylation products of the human TRMT10C-SDR5C1 methyltransferase complex. (**A–C**) The G9 methylation product is 1-methylguanosine (m^1G). (**A**) Position 9-labeled (mt)tRNA^{Ile} was methylated with recombinant TRMT10C-SDR5C1 and the RNA hydrolysate resolved by two-dimensional TLC. Origin and direction of migration in solvent A and B are indicated. Guanosine monophosphate (G) and its methylated derivative (m^1G) were identified by comparison to reference maps (24). (**B**) (Mt)tRNA^{Ile} was methylated with recombinant yeast Trm10p and the RNA hydrolysate resolved by two-dimensional TLC. (**C**) RNA hydrolysates from (**A**) and (**B**) were mixed and resolved by two-dimensional TLC. (**D**) The methyl group is derived from *S*-adenosyl methionine (SAM). Unlabeled, *in vitro* transcribed (mt)pre-tRNA^{Ile} (250 nM) was incubated with 50 nM recombinant TRMT10C-SDR5C1 and 10 µM *S*-adenosyl [methyl-^{14}C]-methionine ([^{14}C] SAM). Samples were removed at the indicated time points and resolved by denaturing PAGE (lanes 1-3). A ^{32}P-labeled (mt)pre-tRNA^{Ile} was loaded as a size marker (lane 5). (**E** and **F**) The A9 methylation product is 1-methyladenosine (m^1A). (**E**) Position 9-labeled (mt)tRNA^{His} was methylated with recombinant TRMT10C-SDR5C1 and the RNA hydrolysate resolved by two-dimensional TLC. Origin and direction of migration in solvent A and B are indicated. Adenosine monophosphate (A) and its methylated derivative (m^1A) were identified by comparison to reference maps (24). (**F**) A sample of the RNA hydrolysate from (**E**) was treated with ammonia to induce the isomerization of m^1A to m^6A (Dimroth rearrangement; ref. 27) and subsequently resolved by two-dimensional TLC. Adenosine monophosphate (A), its methylated derivative (m^1A) and the latter's isomer m^6A were identified by comparison to reference maps (24).

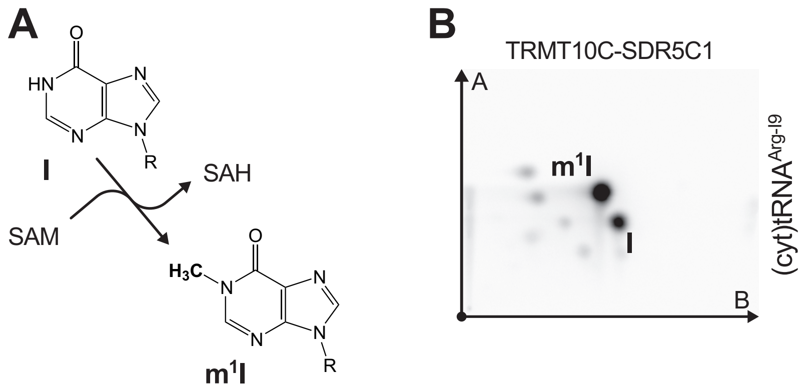

Supplementary Figure S3 Inosine methylation by the human TRMT10C-SDR5C1 methyltransferase complex. (**A**) Enzymatic methylation of the N^1 of inosine using *S*-adenosyl methionine (SAM) as the methyl group donor and release of *S*-adenosyl homocysteine (SAH). (**B**) The I9 methylation product is 1-methylinosine (m^1I). Position 9-labeled (cyt)tRNA$^{Arg\text{-}I9}$ (G9 replaced by inosine) was methylated with recombinant TRMT10C-SDR5C1 and the RNA hydrolysate resolved by two-dimensional TLC. Origin and direction of migration in solvent A and B are indicated. Inosine monophosphate (I) and its methylated derivative (m^1I) were identified by comparison to reference maps (24).

SUPPLEMENTARY REFERENCES

18. Rossmanith, W. (2012) Of P and Z: Mitochondrial tRNA processing enzymes. *Biochim. Biophys. Acta*, **1819**, 1017-1026.
24. Grosjean, H., Droogmans, L., Roovers, M. and Keith, G. (2007) Detection of enzymatic activity of transfer RNA modification enzymes using radiolabeled tRNA substrates. *Methods Enzymol.*, **425**, 55-101.
27. Jones, J.W. and Robins, R.K. (1963) Purine nucleosides. III. Methylation studies of certain naturally occurring purine nucleosides. *J. Am. Chem. Soc.*, **85**, 193-201.
43. Gobert, A., Gutmann, B., Taschner, A., Gößringer, M., Holzmann, J., Hartmann, R.K., Rossmanith, W. and Giegé, P. (2010) A single *Arabidopsis* organellar protein has RNase P activity. *Nat. Struct. Mol. Biol.*, **17**, 740-744.
46. Persson, B., Kallberg, Y., Bray, J.E., Bruford, E., Dellaporta, S.L., Favia, A.D., Duarte, R.G., Jörnvall, H., Kavanagh, K.L., Kedishvili, N. *et al.* (2009) The SDR (short-chain dehydrogenase/reductase and related enzymes) nomenclature initiative. *Chem. Biol. Interact.*, **178**, 94-98.
